# Does the B7-H3 Immune Checkpoint Have High Potential as a Therapeutic Target?

**DOI:** 10.3390/cells15030239

**Published:** 2026-01-26

**Authors:** Marco Agostini, Pietro Traldi, Mahmoud Hamdan

**Affiliations:** Istituto di Ricerca Pediatrica Città della Speranza, Corso Stati Uniti 4, 35100 Padova, Italy; m.agostini@unipd.it (M.A.); mhglaxo@gmail.com (M.H.)

**Keywords:** B7-3H immune checkpoint, immune cell inhibitors (ICIs), immune-related adverse events, mass spectrometry-based proteomics

## Abstract

B7-H3 (CD276), a member of the B7 family of proteins, is known to play a key role in the progression of a number of cancers. This protein is selectively expressed in both tumor cells and immune cells within the tumor microenvironment. Various investigations, including a number of clinical trials, have reported high levels of expression of this protein in cancerous tissues compared to their healthy counterparts. This difference in expression attracted various research efforts to establish whether such a difference can be linked to the therapeutic potential of this molecule. It is worth noting that B7-H3 is not the only immune checkpoint expressed at different levels in cancerous and healthy cells. Therapeutic strategies, based on different levels of expression, have been tested with other checkpoints. To inhibit the expression of some checkpoints, immune checkpoint inhibitors (ICIs) were developed. The introduction of these inhibitors for the treatment of some forms of advanced-stage tumors has been justly described as an important milestone in the landscape of immune therapy. Years after the launch of these inhibitors, numerous clinical trials revealed that these inhibitors benefit a narrow subset of patients suffering from advanced-stage tumors, while the majority of patients treated with these inhibitors either did not respond positively or simply did not respond at all (refractory patients). Other clinical trials showed that this form of treatment can provoke serious immune-related adverse events (irAEs). It is fair to state that changes in the expression level of a given protein in diseased tissue is an important parameter to take into account in the assessment of such a protein as a therapeutic target. However, the last ten years have demonstrated that taking the level of expression of a given checkpoint within a cancerous tissue is not sufficient to consider such expression a reliable predictive biomarker for the investigated disease. On the other hand, to establish a solid base for a given therapeutic strategy, these varying levels of expression have to be combined with a deep understanding of the biology of the molecule under investigation, as well as the identification and thorough analysis of the relevant signaling pathways, particularly those communicating with both the investigated molecule and the immune system. Recently, a number of pharmaceutical and biotechnology firms have suggested that B7-H3 is a highly promising therapeutic target for the development of immune therapeutics. In this review, we ask why hopes of better therapeutic performance are attached to this immune checkpoint. A partial answer to this question is provided through the careful consideration of the available data generated by various clinical trials. The contribution of mass spectrometry-based proteomics to this area of research is highlighted.

## 1. Introduction

Blocking certain inhibitory receptors in the immune system through the use of immune checkpoint inhibitors (ICIs) still represents the main component of immunotherapy for a number of tumors, particularly those in the advanced stage. Numerous studies, including several clinical trials, have shown that the positive response rate among patients receiving immune checkpoint-targeting drugs remains disappointingly low [1,2]. The same studies have demonstrated that the majority of patients undergoing such treatment tend to develop acquired resistance [3,4,5] to the therapy as well as a high risk of developing serious immune-related adverse events (irAEs) [6,7,8,9]. Currently, there are intense research efforts to develop more efficient checkpoint inhibitors that can benefit a wider population of patients, which at the same time have an acceptable risk of causing adverse events. It can be said that such research activities can draw some useful lessons from earlier experiences to discover and develop inhibitors of other classes of proteins implicated in resistance to therapy. Years prior to the introduction of ICI therapy, intensive research activities were dedicated to the discovery of the inhibitors of transmembrane proteins known as ATP-binding cassette (ABC) transporter proteins [10,11]. There are 48 known members of this family [10]. P-glycoprotein (also known as ABCB1 and MDR1) was the first identified mammalian ABC transporter protein and the most investigated. Numerous investigations have demonstrated that the overexpression of this protein in cancerous cells was one of the reasons for poor response to chemotherapy in many cancer types [12,13]. Unfortunately, over 40 years of research failed to produce anything that could be described as an effective and specific inhibitor. During this long period, many potential inhibitors were extensively investigated, but none of them obtained the approval of either the FDA (Food and Drug Administration) or the EMA (European Medicines Agency). This disappointing outcome has been attributed to many factors, including the attribution of drug resistance to a single mechanism (in this case, the overexpression of p-glycoprotein), when numerous studies have demonstrated that a multiplicity of mechanisms are behind such resistance [14,15]. A similar scenario has been witnessed in certain research activities regarding the search for immune checkpoint inhibitors and their potential in new therapeutic strategies. For example, the overexpression of one of these proteins in cancerous tissues compared to their healthy counterparts is enthusiastically treated as a strong indication for a therapeutic target and/or as a predictive biomarker of the investigated tumor. It is fair to say that the overexpression of a given immune checkpoint in diseased tissues should be considered as one of the indications of some correlation between the level of expression and the investigated tumor. However, such indication on its own is not sufficient to describe such immune checkpoint as a potential therapeutic target for the investigated disease.

### 1.1. Potential Contribution of Liquid Biopsies to Problem Resolving in Cancer Research

**Figure 1:**
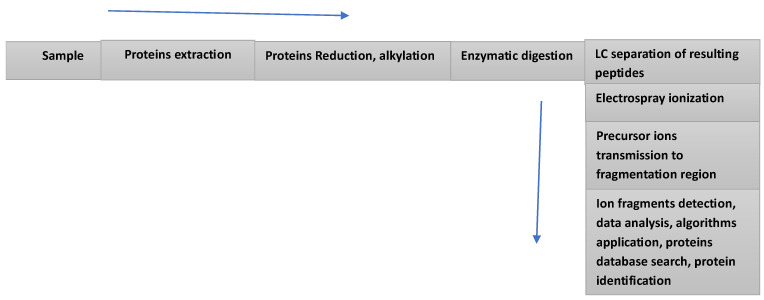
Schematic representation of a workflow commonly used in the analysis of protein mixtures. This experimental arrangement can be used in three different modes of analysis, depending on the sample to be analyzed: “bottom-up” method for a digest of short peptides (5–20 amino acids); middle-down (20–50 amino acids); and top-down (intact small proteins). The main difference between the three methods is limited to sample preparation and the choice of the LC components.

It can be said that the various investigations into B7-H3 have benefited from a more diffused clinical application of liquid biopsy (body fluids) and mass spectrometry-based proteomics. In recent years, implementing liquid biopsies into clinical decision making made a notable contribution to the speed of analysis and the design of important clinical trials, which require serial sampling, which was not practical with the more established tissue sampling [16,17]. An essential component in the search for efficacious immune therapeutics against various forms of cancer is the monitoring of the cellular immune response over a relatively long period of follow-up. The more frequent use of biofluids in clinical analysis has provided a source of essential information on immune responses, which was not possible through the use of tissue sampling. High throughput biofluids analysis can provide much needed information at the cellular, DNA, RNA, epigenetic, protein, and metabolome levels. Such information is essential for the success of clinical trials, designed to investigate the immune response to potential therapeutic target such as B7-H3 immune checkpoint. In liquid biopsy (LB) sampling, peripheral blood, serum/plasma, and other biofluids can be collected at different time points prior, during and after treatment. Such longitudinal sampling allows for almost real-time monitoring of the response to the treatment, and the early detection of irAEs, which may manifest during and after treatment. Another relatively recent development in the analysis of immune checkpoints is the more clinical use of MS-based proteomics [18,19,20]. The combination of liquid chromatography with mass spectrometry (LC/MS) (Figure 1) has demonstrated that such a combination can provide solutions for relevant problems in oncology. The following example is not directly related to B7-H3; however, it demonstrates the value of the application of LB/MS in medical problem resolving.

### 1.2. Detection of Measurable Residual Disease (MRD) in Multiple Myeloma

The last few years have witnessed advances in the detection of measurable residual disease (MRD) in multiple myeloma. Despite these advancements, some patients who were examined with sensitive techniques such as multi-parametric flow cytometry (MFC). and next-generation sequencing (NGS) still experienced disease progression [21]. To evaluate the detection of MRD by different techniques, the authors compared the sensitivity of peripheral blood (PB)-based methods with bone marrow (BM)-based methods, including positron emission tomography/computed tomography (PET/CT), serum protein electrophoresis (SPEP), and immunofixation (IFIX). In their study, the authors enrolled 76 patients, newly diagnosed with secretory multiple myeloma (MM). Liquid chromatography/mass spectrometry (LC-MS) and matrix-assisted laser desorption ionization time-of-flight mass spectrometry (MALDI-TOF MS). These measurements were part of multicenter phase II study to assess the efficacy and safety of three anti-MM drugs [22]. The results of the above study were supported by a more recent investigation. In this recent study, MALDI-TOF MS was used to detect MRD in PB serum samples derived from newly diagnosed patients with multiple myeloma [23]. Serum samples were obtained from 138 multiple myeloma patients undergoing maintenance therapy. It is well known that multiple myeloma (MM) is characterized by the excessive production of monoclonal immunoglobulins (M proteins). MS analysis of liquid biopsy samples, such as serum, has been demonstrated as a highly suitable method for the routine screening of M proteins, detecting low levels of M proteins produced by residual tumor cells, i.e., minimal residual disease (MRD). Both studies cited here concluded that blood-based MS analysis and bone marrow-based methods can be considered two complementary approaches for the detection and assessment of MDR.

The reader may ask why the choice of an example on the use of MS-based analysis to resolve a problem is not directly related to the investigation of B7-H3. The following considerations may give a partial answer to this question: (i) The detection of MDR still represents one of the big challenges in the world of oncology. It occurs in all types of cancer without exclusion; it is one of the problems encountered daily in hospitals and requires fast and accurate outcomes, and detection errors can be devastating for patients and their family. (ii) Mass spectrometry is still struggling to gain its deserved space in hospitals as an analytical tool to perform various analyses alongside more established analytical tools. (iii) The difficulty of accepting MS as a routine method of analysis is not limited to hospitals. Taking the case of immune checkpoints, including B7-H3 as an example, there is a general agreement that mass spectrometry-based proteomics is the most suitable technique for proteins analyses, particularly those present in complex biological samples; such analysis can provide indispensable information on structure, post-translational modifications (PTMs), and conformation in liquid, protein/protein and protein/legend interactions. We have to remind ourselves that a number of post-translationally modified immune checkpoints, including B7-H3, are in the radar of big pharmaceutical industries as therapeutic targets for various pathologies. That said, the use of MS-based proteomics towards these efforts remains very limited.

It is well established that B7H3 is expressed in various solid tumors and has been considered as an attractive target for cancer therapy [24]. In this text an attempt is made to discuss recent research efforts aimed at exploring the potential of B7-H3 as a therapeutic target, and the development of novel B7-H3 targeting antibody–drug conjugates.

## 2. Discussion

### 2.1. Structure and Modifications of B7-H3 (CD276)

**Figure 2:**
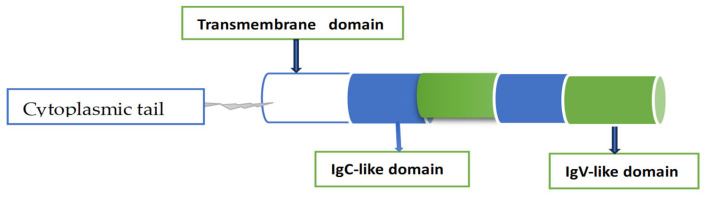
Schematic representation of 4Ig B7-H3 isomer. The other isomer, 2IgB7-H3, has a single pair of (IgC)-like domains (blue) and (IgV)-like domains (green). Some of the structural information in Figure 2 is based on the data in [26].

**Figure 3:**
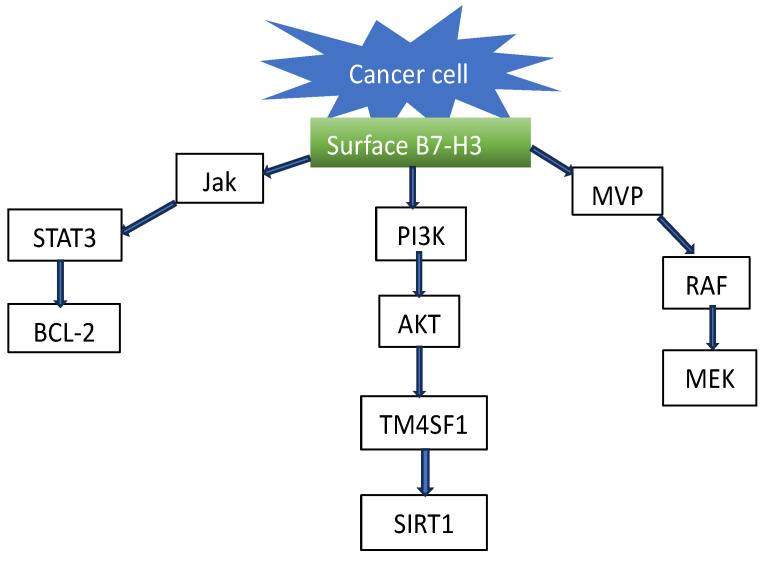
Some signaling pathways, which can be triggered by B7-H3 expressed on cancer cell membrane The figure is partially based on the data reported in [26].

B7-H3 is a member of the B7 family, which contains other nine members: B7-1 (CD80), B7-2 (CD86), B7-H1 (PD-L1), B7-DC (PD-L2), B7-H2 (ICOSLG), B7-H4 (VTCN1), B7-H5 (VISTA), B7-H6 (NCR3LG1), and B7-H7 (HHLA2). They possess approximately 20% amino acid sequence identity among them [25]. B7-H3 has two isoforms, 4Ig B7-H3 and 2Ig B7-H3. The human-dominant 41g isoform encodes 534 amino acids and consists of two identical pairs of IgV-like and IgC-like domains. The minor 21g isoform (mouse) includes a single pair of IgV-like and IgC-like domains. Figure 2 shows a schematic representation of the dominant 41g B7H3 isoform [26]. Various investigations have demonstrated that the expression of B7-H3 in different types of cancer can be regulated by various modifications. Post-transcriptional micro-RNA was found to be responsible for a regulatory mechanism, affecting the expression of this protein [27,28]. This mechanism was tested in breast cancer; the authors monitored 20 different micro RNAs, and found that 13 of them were bound to B7-H3 [29]. The impact of micro-RNA on the expression of this protein has been reported for different types of cancer, including multiple myeloma, colorectal cancer cells, and head and neck squamous cell carcinoma. In a relatively recent study, it was demonstrated that microRNA (miR-29a, miR-29b, and miR-29c) targeted B7-H3, expressed in neuroblastoma cells, resulting in NK cell activation and NK-mediated cytotoxicity, thereby inducing an anti-tumor immune response through the interaction of mir29 with neuroblastoma cells [30,31]. In addition to regulating the immunological microenvironment, B7-H3 has been reported to activate a number of signaling pathways, including ERK, PI3K, and Stat3 in cancer cells (see Figure 3).

B7-H3 is known to experience extensive glycosylation and, to a lesser extent, phosphorylation and ubiquitination [32]. N-glycosylation is the main post-translation modification (PTMs), affecting seven asparagine residues (N91, N104, N189, N215, N322, N407, and N433) [33,34]. As well as this extensive glycosylation, two other modifications have been reported: phosphorylation at serine (S513) and threonine (T515), and ubiquitination at two lysine residues (K521 and K526). Although B7-H3 is considered a highly promising therapeutic target, the absence of known receptors for this protein renders this checkpoint a highly challenging pharmaceutical target. That said, the identification and characterization of key PTMs of the same protein may pave the way for the development of blocking antibodies targeting modified B7-H3 localized at N91/309 and N104/322. It is useful to take into consideration that extensive N-glycosylation has been reported for other immune checkpoints. PTMs have been demonstrated to impact the expression of PD-L1 on tissues, and such expression is considered the main predictive biomarker in response to anti-PD-1/PD-L1 therapy [35,36]. Although a number of investigations have demonstrated the impact of glycosylation on the immune response of B7-H3, a deeper understanding of this modification and its influence on the various functions of this protein remains fragmentary.

### 2.2. Some PTMs Experienced by B7-H3

Protein glycosylation is acknowledged as one of the major post-translational modifications; proteins can undergo glycosylation during and/or after translation. This key modification is known to impact cancer progression by influencing important biological processes such as protein folding, degradation, cellular localization, immune modulation, and intercellular signaling. Altered glycosylation patterns are increasingly considered to be important drivers of tumorigenesis [37,38,39]. It is interesting to observe that tumor cells often display a wide range of glycosylation alterations compared with their non-transformed counterparts, and these alterations play a critical role in the development and progression of cancer [40,41,42]. To emphasize the important role of post-translationally modified B7-H3, we choose to discuss a number of examples, which have dealt with this argument.

In a fairly recent study, it was demonstrated that N-glycosylation of B7-H3 at specific asparagine residues (N) can induce strong anti-tumor immunity. To clarify the role of specific glycosylated asparagine residues that mediate the function(s) of B7-H3, the authors have used a battery of analytical techniques, including LC/MS-MS, immunofluorescence, immunoblot and immunoprecipitation, quantitative real-time PCR, and immunohistochemical staining. This investigation provided important clarifications regarding the impact of two pairs of glycosylated asparagine, N91/309 and N104/322, on the activities of modified B7-H3. These activities include protein trafficking to the cell surface membrane and structural stabilization, which prevents the likely degradation of this protein. According to the authors, their study provided evidence that the glycosylation of the two pairs of asparagine was essential to the capability of B7-H3 to inhibit T cell proliferation and activation.

In an earlier study, the same research group investigated the role of N-glycosylated B7-H3 protein stabilization and immunosuppression in patients with triple-negative breast cancer (TNBC) [26]. TNBC is a heterogeneous subtype of breast cancer that generally has a poor prognosis, with high rates of systemic recurrence or metastatic potential and refractoriness to conventional therapy. TNBC was also found not to respond to anti-PD-1/PD-L1 therapy [43]. To obtain a deeper understanding of B7-H3 upregulation, the authors examined over 110 pairs of breast cancer tissues. The generated data showed that B7H3 mRNA expression in breast cancer tissues was actually significantly higher than that in matched normal breast tissues. The authors also performed Kaplan–Meier meta-analyses, using the online Kaplan–Meier–Plotter breast cancer database and the database retrieved from Breast Cancer Gene-Expression Miner [44,45]. According to the authors, this analysis showed that a high expression of B7H3 mRNA was associated with poor recurrence-free survival (RFS) and poor early distant metastasis in patients with basal tumors but not luminal and HER2 tumors. As mentioned earlier in this text, the absence of known receptors for B7-H3 renders this checkpoint a highly challenging pharmaceutical target. The development of monoclonal antibodies to target specific glycosylation sites can be considered an attempt to bypass such difficulty. Furthermore, both the studies cited here underline the important role of N-glycosylation in anti-tumor immunity.

Before discussing the role of B7-H3 as a prognostic biomarker for certain cancer types, it is relevant to refer to a recent work, which proposes the de-glycosylation of this checkpoint as an alternative therapeutic strategy to antibodies targeting specific glycosylation sites [46]. This work argues that N-glycosylation plays a central role in its stability and interaction with immune cells; therefore, removing the N-glycans can be considered an alternative therapy to the main therapeutic approach, where these glycans are targeted with antibodies. This hypothesis has a number of serious limitations. First, there are no published studies to indicate the exact composition of B7-H3, expressed on different cancerous cells. In other words, the percentages of intact and glycosylated B7-H3. This is a relevant observation, because we still do not have enough information on the specific role of each of the two components in the various functions of this protein. Second, de-glycosylation was experimented with another immune checkpoint over six years ago [47]; the scope of the experiment was not therapeutic, but to enhance PD-L1 detection and to predict anti-PD-1/PD-L1 therapeutic efficacy. The authors described a protocol to eliminate glycosylation prior to the immunohistochemistry (IHC) staining of the tissues under investigation. The potential of de-glycosylation of B7-H3 to form part of a new therapeutic strategy needs to be supported by further studies, including well-designed clinical trials involving the high numbers of patients suffering different forms of cancer.

### 2.3. B7-H3 as Prognostic Biomarker

High expression of B7-H3B7 has been reported in different cancer types—association between this high expression and poor prognosis has been established for some time (see Table 1). This aberrant upregulation has rendered this checkpoint a highly attractive target as prognostic biomarker, and as a highly promising immunotherapeutic target for a number of cancer types. The evaluation of B7-H3 as a diagnostic, prognostic biomarker and its association with the clinical outcome for various forms of cancer has been discussed in a number of recent studies. In a retrospective study involving one hundred patients with confirmed histopathological low-grade and high-grade gliomas, B7-H3 expression was assessed using immunohistochemistry [48]. This study concluded that the level of expression of this checkpoint could differentiate between the two subtypes of gliomas. According to the same study, patients with high B7-H3 had significantly shorter overall survival (median 6 months vs. 42 months) and progression-free survival (median 3 months vs. 25 months). Based on these conclusions, the authors described the high expression of B7-H3 as a reliable biomarker for differentiating between the two subtypes of tumor, and a biomarker which is associated with poor survival outcome. These highly optimistic conclusions need to be further confirmed through the use of much higher number of patients within large clinical trials. This study compared the level of expression of B7-H3 in high and low gliomas and did not report a comparison between the expression level in diseased tissues and their healthy counterparts. The absence of such a comparison renders the analysis of the expression level in diseased tissues less informative.

Immunohistochemistry (IHC) and Western blotting were used to examine tissue microarrays obtained from 268 gastric cancer (GC) patients who underwent surgeries. The co-expression of B7-H3 and an antiphagocytic protein, CD47, was assessed as a predictive biomarker for the poor prognosis of GC patients. The authors reported a significant correlation between high expression of B7-H3 and CD47 and poor overall survival in gastric cancer patients. The correlation between the high-level expression of B7-H3 in gastric cancer patients was investigated in a more recent study [49]. In this study, IHC was used to assess the expression levels of B7-H3, CD39, and CD8 in 268 GC tissues and 80 gastric precancerous lesions. Multiplex IHC was used to determine the co-localization of B7-H3 and CD39 in GC tissues. Kaplan–Meier survival analysis and Cox regression models were applied to evaluate clinical outcomes in different GC subgroups. This study reported a number of conclusions. Both B7-H3 and CD39 expression showed a stepwise increase during gastric carcinogenesis including low-grade and high-grade intraepithelial neoplasia, and a significant positive correlation was observed between B7-H3 and CD39 expression. The expression of both proteins was found to be significantly correlated with tumor volume, tumor stage, tumor depth, lymph node metastasis, lymph node involvement, and distant metastasis. The three studies cited above have a number of common elements responsible for what can be described as the common limitations of these studies. These common elements include the use of tissue samples rather than biofluids, the use of IHC for the assessment of the level of expression of the investigated proteins, and the correlation of such levels with the investigated pathology.

**Table 1:** Some cancer forms in which high expression of B7-H3 have been reported.

Type of Cancer	Interpretation of B7-H3 Overexpression in Cancerous Tissues	Ref.
Colorectal carcinoma (CRC)	In CRC, B7-H3 promotes tumor angiogenesis through the NF-κB pathway, and upregulation of B7-H3 increases the expression of intracellular TNF-α, which modulates the inflammatory response and promotes tumor growth by inducing cell survival.	[50,51]
Breast cancer	B7-H3 expression was found to be positively correlated with high number of tumor-infiltrating lymphocytes in breast cancer. High expressions of B7-H3 can promote breast cancer cell proliferation and the brain metastasis of breast cancer.	[52]
Lung cancer	The level of B7-H3 in lung cancer cells was found to be positively correlated with the number of monocytes/macrophages. B7-H3 promotes the expression of HIF-1α by upregulating the phosphorylation levels of NF-κB, enhancing the anti-apoptotic ability of monocytes/macrophages and facilitating their aggregation in the tumor microenvironment.	[53,54]
Gastric cancer	B7-H3 modulates the metabolism of glutathione to increase the stemness of gastric cancer cells via the AKT/pAKT/Nrf2 signaling pathway. Granulocyte–macrophage colony-stimulating factor is initially produced in gastric cancer cells, activating the Jak2/Stat3 signaling pathway to mediate the activation of tumor-associated neutrophils and the expression of B7-H3.	[55,56]
Acute myeloid leukemia (AML)	Inhibiting B7-H3 expression in AML patient samples enhanced NK cell-mediated apoptosis in AML cells, thereby promoting AML cell death and extending OS in AML patients. B7-H3 has the potential to serve as a prognostic marker for AML.	[57,58]
Glioblastoma (GBM)	B7-H3 expression was significantly elevated in GBM tissues relative to normal tissues, with studies indicating a negative correlation between B7-H3 levels and overall survival (OS).	[59,60]

To understand why such common elements between the three studies are responsible for the main limitations of the same studies, the following considerations may help: First, the use of tissue samples rather than liquid biopsy samples renders longitudinal analysis extremely limited. This limitation reduces the efficient monitoring of the prognosis and progress of the investigated disease. Second, the use of IHC to assess the expression of PD-L1 on cancerous tissues has demonstrated that such expression does not reflect the heterogeneity of the monitored disease. The heterogeneity of PD-L1 expression on the investigated tissues had an impact on the predictive reliability in response to ICIs therapy. These considerations are not intended to undermine the clinical and the scientific value of these studies; on the contrary, these considerations are intended to draw attention to the fact that the use of liquid biopsy sampling can add further value to the results generated by tissue sampling.

## 3. B7-H3 as an Immunotherapy Target

Twenty-five years after its discovery, B7-H3 is still the orphan of a definite receptor. Despite this pharmaceutical hurdle, various research activities and preclinical studies have demonstrated that targeting B7-H3 as an inhibitory immune checkpoint in preclinical models could suppress tumor growth [61,62]. The upregulation of B7-H3 expression in various forms of cancer (see Table 1) and the correlation between such upregulation and poor prognosis have rendered this immune checkpoint a highly appetizing therapeutic target, particularly for pharmaceutical companies and small biotech. Over the last 10 years there have been intense research efforts to discover and develop effective B7-H3-targeting molecules. Such efforts have included the investigation of monoclonal antibodies, bispecific antibodies, and more recently, antibody–drug conjugates (ADCs). Clinical trials with monoclonal and bispecific antibodies have demonstrated limited success, which has shifted a major attention to antibody–drug conjugates [63,64,65].

### 3.1. Drug-Conjugate Antibodies Targeting B7-H3

**Figure 4:**
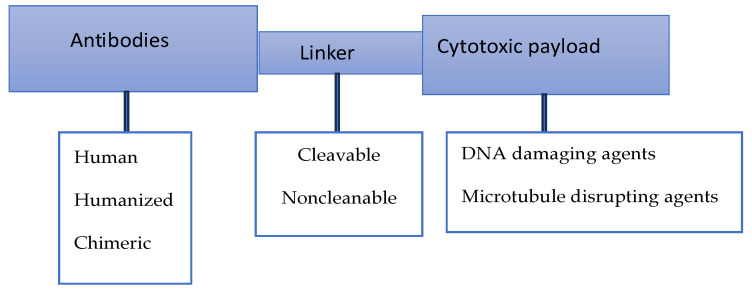
Schematic representation of the main components of an antibody–drug conjugate (ADC). DNA damaging agents may include calicheamicins, duocarmycins, or DXd (topoisomerase I inhibitor), while the disrupting agents may include auristatins or maytansinoids. This figure is partially based on information in [67].

The introduction of antibody–drug conjugates (ADCs) into the arena of antibodies is considered a success story in the search for effective molecules to target various immune checkpoints. Basically, these structures combine the specificity of monoclonal antibodies with a wide range of cytotoxic agents, capable of selectively delivering toxic drugs to target antigen-expressing tumor cells (Figure 4). Various linkers are used to link the antibodies with cytotoxic drugs [66,67]. These molecules have demonstrated a respectable efficacy in cancer treatment; however, the development of adverse events remains one of the main challenges in this innovative treatment. More specific details of these events are discussed in the next section of this review. The data generated by a number of clinical trials in which B7-H3 has been targeted by a number of ADCs indicate highly promising therapeutic potential for the treatment of solid tumors. To gain more information on such potential, the results of some of these trials are considered here. Table 2 provides a very short list of the trials in which ADCs are used to target B7-H3. It has to be said that the list of ongoing trials is much longer, and no single work can address such a list. The multiplicity of the ongoing clinical trials targeting B7-H3 is a reflection of the confidence that the molecule carries a high therapeutic potential in the landscape of immunotherapy.

### 3.2. Mechanism of Action of Antibody–Drug Conjugates (ADCs)

The current literature suggests that over the last 25 years, marketed ADCS have been the results of three distinct phases of development [71]. Conjugates of the first phase had a narrow therapeutic window due to the weak toxicity in the payload, unstable structure, and the easy detachment of the toxin [72]. In the second phase, ADCs used humanized monoclonal antibodies and more potent cytotoxic drugs, resulting in improved efficiency and a wider therapeutic window compared to the earlier generation [73,74]. In the most recent generation (third phase), these molecules contain more site-specific conjugates, resulting in more enhanced uniformity of the drug-to-antibody ratio, reduced adverse events, increased efficacy, and a wider therapeutic window compared to previous generations [75]. The identification and eventual understanding of the mechanism(s) of action of a potential therapy are central in the success or failure of the therapy. Understanding the mechanism of action behind targeting B7-H3 with antibody–drug conjugates entail a careful and deeply informed selection of the three main components of the conjugate under investigation. This informed selection is supported by the recent literature, which shows that over 300 ADCs have been explored for various tumor indications [76], yet only a dozen of these molecules has been approved by the FDA). The action of the ADCs follows the following sequence. (a) The antibody binds to the target antigen, forming ADC–antigen complex, which is transported into the lysosomes through receptor-mediated endocytosis. (b) This complex is broken down by the acidic environment or the enzymes within the lysosomes. (c) The breakdown of the complex releases the cytotoxic upload, responsible for the elimination of the tumor cells [67]. Numerous clinical trials and various investigations have demonstrated the potential of ADCS as potent therapy for various forms of cancer. These encouraging results include those conjugates targeting B7-H3. It can be said that these molecules have demonstrated their therapeutic efficacy; however, there remains the challenge of the serious adverse events associated with these molecules.

### 3.3. Adverse Events Associated with Drug-Conjugate Antibodies

The use of antibody–drug conjugates is justly considered one of the success stories in the treatment of various forms of solid tumors. Over the last decade, the FDA has approved a number of these molecules for the treatment of solid tumors (see Table 3 and Table 4). Although there are significant differences between various ADCs, the basic construction remains the same. Each of these molecules can precisely deliver a pay load of toxic agent to a specific diseased site. Various investigations, including clinical trials, have demonstrated their safety and efficacy in treating solid tumors. Other studies have shown that the adverse events associated with each of these molecules remains some of the main challenges facing this innovative therapy [77,78,79,80]. In this text there is no room for a detailed discussion regarding the adverse events of ADCs; however, the following observations are of relevance to the argument of this text. (i) An important chapter in a clinical trial, seeking approval for a given molecule, is the precise characterization of the safety profile of the potential drug under investigation. The safety profile includes a predictive identification of likely adverse events, which may be associated with a drug candidate. It is no exaggeration to state that the interruption of the majority of the clinical trials is normally associated with the poor safety profile of the investigated drug. Table 3 provides a short list of some of the adverse events associated with approved ADCs for the treatment of various forms of cancer, while Table 4 gives a longer list of approved ADCs and their indications as therapy against various forms of cancer. (ii) The official description of the trials listed in Table 2 describes the assessment of efficacy of the tested ACDs as the primary objective of the trial, as well as the safety profile, including the identification of AEs as a secondary objective. This description may suggest that the priority of such trials is to find an efficacious drug first, while establishing the safety profile of the same drug is a second priority. The experiences of immune-related adverse events (irAEs) associated with immune checkpoint inhibitors (ICIs) tells us that establishing the precise safety profile of a given drug should be considered the top priority in the course of a potential drug. The introduction of ICIs has revolutionized immunotherapy; however, unfortunately, because of the associated irAEs, such a revolution has only benefited a small subset of patients. The main reason for such a disappointing outcome is the failure to obtain a precise picture of the associated adverse events, which, for the majority of advanced cancer patients, has meant the interruption of ICIs therapy. To address this serious limitation of ICIs, intense research efforts have focused on the discovery of effective biomarkers capable of the early identification of patients who are likely to draw a significant therapeutic benefit from ICIs therapy without developing serious irAEs. These predictive biomarkers should have been investigated and identified in clinical trials investigating the efficacy of ICIs, and not years after their launch.

## 4. Other Immune Therapeutic Strategies

The current literature shows that most published clinical trials are researching the use of antibody–drug conjugates to target B7-H3 as a main therapeutic strategy. That said, there are other therapeutic strategies showing highly promising therapeutic potential. A brief description of some of these strategies is given below.

### 4.1. Chimeric Antigen Receptor (CAR-T)

Recently, CAR-T cell has emerged as a highly promising method within certain therapeutic strategies. This method has shown promising results in a number of preclinical studies. In addition, the initial data from some ongoing clinical trials indicate that the use of this therapy to target B7-H3 can contribute to emerging therapeutic strategies, targeting a number of tumors, including melanoma, glioblastoma, prostate cancer, lung, gynecological, and renal cancers. Other studies have demonstrated promising its therapeutic efficacy against hematological malignancies [98,99]. So far, there are seven FDA-approved CAR T cell therapies available for treating various hematological malignancies. These therapies are indicated for the treatment of specific types of leukemia, lymphoma, and multiple myeloma. The therapeutic strategy based on these therapies is still in the phase of optimization in order to address a number of limitations, including the scarcity of target antigens, severe toxic effects, and the emergence of acquired and intrinsic resistance to therapy. The main limitation of CAR-T therapy remains the difficulty of inducing an immune response. This lack of patients’ immune response is attributed to the single-chain variable fragment (scFv) domain within the structure of CAR-T, which is mainly of non-human origin [100]. More details on the structure and the mechanism of action of CAR-T have been given in a recent review [101].

### 4.2. Natural Killers (NK) and T Cell Engagers

Both T and NK cell engagers are targeted immunotherapies; both therapies are designed to strengthen the immune system through the activation of precise effector cells. The T cell engagers redirect T cells into the proximity of tumor cells with a tumor-targeting domain and trigger T cell signaling via the CD3 crosslinking domain [102,103]. Despite the current limitations of these engagers, including their low efficacy in solid tumors, toxicity, and resistance, unlike conventional chemotherapy and radiotherapy, which can damage healthy tissues, these engagers induce tumor-specific immune responses with little or no off-target effects, making them a promising therapeutic modality [104,105]. It is reasonable to state that T cells and NK cells have a complementary role in tumor immunity; however, they have two different molecular mechanisms of tumor cells recognition [106]. For example, all the key functions of T cells are controlled by the T cell receptor (TCR), including cytotoxicity, cytokine production, and proliferation [107]. T cells identify tumor cells through the TCR-mediated recognition of MHC-bound peptide antigens [107]. This mechanism of tumor cells’ recognition allows tumors to escape immunity by CD8 T cells through the downregulation or loss of MHC-I expression [108]. On the other hand, NK cells use multiple germ line-encoded activating and inhibitory receptors to identify target cells. In other words, the recognition of the tumor cells as well as NK cell-mediated cytotoxicity is controlled by the summing of signals from multiple surface receptors [109]. Currently, there is strong evidence that the central role of T cell-mediated tumor immunity is interconnected with that of NK cells. The molecular mechanisms and the biology of this interconnection are still under investigation.

### 4.3. Targeting B7-H3 in Solid Tumors

Medulloblastoma (MB) is the most prevalent pediatric brain tumor, accounting for nearly 20% of all pediatric brain tumors worldwide [110]. The current treatment options for this devastating disease are not curative and tend to leave the survivors with a poor quality of life. It has to be said that over the last ten years there have been relevant advances regarding the molecular profiling and almost accurate identification of the subgroups of the disease. It can be said that recent advancements in technologies such as next-generation sequencing (NGS) and DNA methylation profiling have played a decisive role in our understanding of the molecular heterogeneity of this disease [111,112,113]. These technologies, together with subsequent validation through transcriptome-based studies, have revealed that MB is composed of at least four major molecular subgroups: wingless/integrated (WNT), sonic hedgehog (SHH), group 3, and group 4, each with its own unique transcriptional, genetic, and clinical characteristics [114,115]. Years of research, extensive clinical trials, and numerous meta studies of various types of cancer have established that gaining a deep understanding of the heterogeneity of a given tumor is the gateway to the correct stratification of patients, effective prognosis, and above all the choice of an efficacious therapeutic regime.

The emergence of chimeric antigen receptor (CAR T) as a novel approach to boost the immune system has given hope to MB patients. Unfortunately, this highly promising therapy has been slowed down by various inhibitory immune checkpoints, including B7-H3. The role of this checkpoint in some solid tumors has been reported in different studies. In one of these investigations, it was reported that the high expression of this protein in cancer stem cells (CSCs) of head and neck squamous cell carcinoma (HNSCC) could contribute to evasion of immune surveillance [116]. According to these authors, the blockage of B7-H3 with monoclonal antibodies eliminated CSCs and inhibited tumor growth and metastasis by enhancing CD8 + T lymphocytes-mediated anti-tumor immunity. It was also reported that CAR T cells directed against B7-H3, expressed in pediatric solid tumors, can also influence non-tumor cell types in the tumor microenvironment (TME) [117]. To apply CAR T cell therapy, it is indispensable to have high-density expression of the targeted antigen (in this case B7-H3). However, to establish sufficient surface expression of this protein, accurate correlation between B7-H3 mRNA is necessary. The existing data suggest that such a correlation is not common in the case of B7-H3, which hampers gene expression measurements that are necessary to establish the targetability of the tumor with B7-H3-directed CAR T therapy. Currently, CAR T immunotherapy demonstrates a high therapeutic potential for the treatment of pediatric solid tumors. This potential role is based on the capability of this approach to target immune checkpoints commonly expressed in various solid tumors. However, there is still a paucity of clinical data to allow a rational assessment of the future role of this innovative technique in the landscape of immunotherapy. This statement is based on a simple comparison (limited to the case of B7-H3) between the current therapeutic activities associated with CAR T cell therapy and those associated with drug-conjugate antibodies targeting B7-H3.

## 5. Conclusions and Future Perspectives

Based on the material discussed in this text regarding the potential of B7-H3 as a highly promising therapeutic target, the following tentative conclusions can be made. First, there is almost a total agreement between numerous investigations that B7-H3 is frequently overexpressed in cancerous cells, while in healthy cells, the expression is at very low levels. The same literature strongly suggests that such overexpression can influence both immune regulation and tumor progression. Other roles attributed to this overexpression include poor prognosis across a wide spectrum of malignancies, metastasis, and resistance to therapy. Whether such roles can be mainly attributed to the overexpression in diseased cells is still strongly debated. Such debate stems from a foggy knowledge of the various mechanisms behind such overexpression. For example, what is the exact composition of the expressed protein (extent of PTMs, level of each isoform, and level of dimerization)? For correct interpretation of the role of B7-H3 overexpression, the bracketed elements have to be seriously considered in clinical trials, investigating the potential of this immune checkpoint as a promising therapeutic target. Second, investigations, including clinical trials of the adverse events associated with ADCs therapy, seem to lag behind investigations that demonstrate the therapeutic efficacy of the same molecules. In other words, the analysis and evaluation of adverse events associated with antibody–drug conjugates (ADCs) therapy seem to lag behind current efforts to demonstrate the efficacy of these molecules in targeting B7-H3. It would be highly beneficial for cancer patients to avoid the scenario encountered with ICIs therapy, where an innovative and efficacious therapy only benefits a small subset of patients, while the rest of the patients cannot receive such therapy because of associated serious immune-related adverse events (irAEs). Third, there are a number of gaps in current efforts to transform B7-H3 from simply a promising therapeutic target to an efficacious therapeutic molecule. These gaps or limitations include the absence of a determined receptor, a lack of deep understandings of the various signaling pathways associated with the overexpression of this immune checkpoint, and the need for more detailed proteomic analyses of the various post-translation modifications and their impact on the functions and activities of this molecule. Emerging therapeutic strategies based on the use of chimeric antigen receptor (CAR-T), and natural killers and T cell engagers may bridge these gaps that have manifested in the therapy based on antibody–drug conjugates.

## Figures and Tables

**Table 2:** Short list of some trials in which ADCs are used to target B7-H3.

Gov. Identifiers of Some Clinical Trials	Objective	Ref.	Relevant Observations
NCT06057922	This trial was designed to evaluate the safety, efficacy, and pharmacokinetics of YL201 in patients with selected advanced solid tumors.	[68]	A multicenter, open-label, Phase 1/2 study. It was started September 2023, the estimated completion date is October 2028, and estimated enrolment is 990 subjects.
NCT06612151	The primary objective of this study is to assess whether treatment with YL201 prolongs overall survival (OS) compared with treatment of topotecan hydrochloride among subjects with relapsed small cell lung cancer (SCLC)	[68]	A Phase III study of YL201 in relapsed small-cell lung cancer. This study was designed to compare the efficacy and safety of YL201 with topotecan hydrochloride in subjects with relapsed small-cell lung cancer (SCLC). The study starts December 2024, its completion is estimated December 2030, and the enrolment is 438 participants (estimated).
NCT06629597	The primary objective of this study was to assess whether treatment with YL201 prolongs overall survival (OS) and increases objective response rate by blinded independent central review compared with treatment of investigator’s choice of chemotherapy among subjects with recurrent or metastatic nasopharyngeal carcinoma.	[68]	A Phase III study of YL201 in recurrent or metastatic nasopharyngeal carcinoma. This study was designed to compare the efficacy and safety of YL201 with the investigator’s choice of chemotherapy in subjects with recurrent or metastatic nasopharyngeal carcinoma who have failed prior PD-(L)1 inhibitor and at least two lines of chemotherapy. started December 2024, with completion estimated December 2028. Its enrolment is 400 (estimated).
NCT04145622	Dose Escalation Part: To evaluate the safety and tolerability, and to determine the maximum tolerated dose and the recommended dose for expansion of ifinatamab deruxtecan (I-DXd). Dose Expansion Part: To investigate the safety, tolerability, and anti-tumor activity of I-DXd when administered as a single agent.	[69]	Study of ifinatamab deruxtecan (DS-7300a, I-DXd) in participants with advanced solid malignant tumors. This is a single group study of participants with advanced solid tumors who have not been cured by other treatments. It is the first time the drug has been used in humans, and consists of two parts. It started November 2019, its completion will be April 2027 (estimated), and its enrolment is 250 participants (estimated).
NCT05280470	This two-part study intends to define the recommended Phase 2 dose of ifinatamab deruxtecan (I-DXd) based on the efficacy, safety, and pharmacokinetics (PK) results observed in participants with extensive-stage small-cell lung cancer (ES-SCLC) who received at least one prior line of platinum-based chemotherapy, and a maximum of three prior lines of therapy (Part 1) and a minimum of two previous lines of systemic therapy (Part 2). This study will also investigate I-DXd anti-tumor activity in this population.	[70]	Ifinatamab deruxtecan (I-DXd) in subjects with pretreated extensive-stage small-cell lung cancer (ES-SCLC). It started March 2022, its completion will be December 2026 (estimated), and its enrolment is 187 particpants.

**Table 3:** Various toxicities associated with ADCs, approved by the FDA.

ADCs	Toxicity	Ref./Observations
Trastuzumab emtansine (T-DM1)/ Trastuzumab deruxtecan (T-DXd)	Thrombocytopenia, including cardiotoxicity, splenomegaly, nodular regenerative hyperplasia, hepatic cirrhosis, and portal hypertension. Myelosuppression, interstitial lung disease, pneumonitis, febrile neutropenia, pneumocystis jirovecii pneumonia, and neutrophil count decreased, and KL-6 increased.	T-DM1 and T-DXd are two promising antibody–drug conjugates for treating advanced HER2-positive breast cancer and HER2-mutated lung cancer [81].
Sacituzumab govitecan (SG), also known as IMMU-132.	The most common AEs of ≥grade 3 were neutropenia (46%), leukopenia (13%), and anemia (8%). Other AEs included meningitis, colitis, and lymphedema collected from the (FAERS) database.	The results, based on six clinical trials involving 1737 patients, were included in the pooled analysis. Part of the results were collected from the FDA Adverse Event Reporting System (FAERS) database [82].
Mirvetuximab soravtansine (MIRV)	The three trials showed low-grade, resolvable gastrointestinal and ocular adverse events (AEs). In total, 50% of patients had ≥1 ocular AEs of blurred vision or keratopathy, the majority being grade ≤2. Grade 3 ocular AEIs occurred in 5% of patients, and one patient (0.2%) had a grade 4 event of keratopathy.	Results based on three trials, involving 464 patients treated with MIRV [83].
Tisotumab vedotin (TV),	A number of adverse events (AEs) were reported, including ocular, peripheral neuropathy, and bleeding. Ocular AEs were most commonly conjunctivitis, dry eye, ulcerative keratitis, blepharitis, and keratitis.	These AEs were initially observed in the phase I/II first-in-human study of tisotumab vedotin (innovaTV 201; NCT02001623) [84].
Enfortumab vedotin (EV)	The analysis identified 5359 reports associated with the EV subgroup; most reports were associated with dermatologic (38.6%), neurologic adverse events (16.5%), or adverse laboratory assessments (19.4%).	The authors employed Bayesian disproportionality analysis based on the information component (IC) to explore the safety profile associated with EV [85].

**Table 4:** Drug conjugates (ADCs) approved by the FDA, and indications for use against various forms of cancer.

Antibody–Drug Conjugates (ADCs)	Indications for Tumor Therapy	Observations/Ref.
Gemtuzumab Ozogamicin (also known as CMA-676).	This received accelerated approval for the treatment of older patients with relapsed CD33-positive acute myeloid leukemia (AML). It was withdrawn in 2010 for safety reasons.	Humanized anti-CD33 monoclonal antibody linked by an acid-labile hydrazone cleavable linker to the cytotoxic agent [86,87].
Brentuximab Vedotin (BV).	The FDA provided accelerated approval in 2011, with two indications. First, for patients with Hodgkin lymphoma, who either relapsed after two or more prior lines of therapy. The second indication was for the treatment of patients with systemic anaplastic large-cell lymphoma after the failure of at least one prior multi-agent chemotherapy regimen.	A CD30-directed ADC consisting of the chimeric IgG1 antibody cAC10, specific for human CD30, and a microtubule-disrupting agent with a cleavable linker [88].
Ado-Trastuzumab Emtansine	In 2013, this molecule was approved for patients with metastatic HER2-positive breast cancer, who had previously received trastuzumab and a taxane. In 2019, the FDA approved TDM1 as a single agent for the adjuvant treatment of patients with HER2-positive breast cancers that had residual disease after receiving neoadjuvant trastuzumab-based therapy.	A HER2-targeted antibody–drug conjugate. The antibody component is a humanized anti-HER2 IgG1 and trastuzumab, and the small molecule cytotoxin is DM1. The linker is non-cleavable [89].
Inotuzumab Ozogamicin	In 2017, this molecule was given FDA approval for the treatment of adults with relapsed or refractory B-cell precursor acute lymphocytic leukemia (ALL).	Inotuzumab ozogamicin comprises an anti-CD22 humanized monoclonal antibody that is linked to calicheamicin, a cytotoxic antibiotic by an acid-labile hydrazone linker [90].
Polatuzumab Vedotin Piiq	This received accelerated approval in 2019 for the treatment of relapsed or refractory diffuse large B-cell lymphoma after receiving two or more therapies.	Polatuzumab vedotin is a conjugate composed of an anti-CD79b monoclonal antibody, linked via a protease-cleavable linker to monomethyl auristatin, which is a potent microtubule inhibitor [91].
Enfortumab Vedotin (EV)	In 2019, EV was granted accelerated approval by the FDA for use in adult patients with locally advanced or metastatic urothelial carcinoma who had previously received treatment with either a programmed death receptor-1 (PD-1) inhibitor or a programmed death ligand receptor-1 (PD-L1) inhibitor, along with a platinum-containing chemotherapy agent.	Enfortumab vedotin (EV) comprises a human monoclonal antibody specific for nectin-4 and linked by a protease cleavable linker with monomethyl auristatin E [92].
Trastuzumab Deruxtecan	T-DXd received accelerated FDA approval in 2019 for patients with unresectable or metastatic HER2-positive breast cancer, who had previously been treated with two or more prior HER2-targeted regimens in the metastatic setting. In 2022, the FDA granted regular approval to T-DXd for patients with unresectable or metastatic HER2-positive breast cancer who had received a prior HER2-targeted regimen in any setting.	T-DXd is composed of an anti-HER2 antibody trastuzumab, a cleavable tetrapeptide-based linker, and a cytotoxic topoisomerase I inhibitor (an exatecan derivative) [93].
Sacituzumab Govitecan	In 2020, sacituzumab govitecan (Trodelvy, Immunomedics) was granted accelerated approval for the treatment of metastatic triple-negative breast cancer in adult patients who previously had received two or more treatments for metastatic disease. A year later, the same molecule was granted regular approval by the FDA for patients with unresectable locally advanced or metastatic triple-negative breast cancer who had previously received at least two prior systemic therapies, with at least one treatment being for metastatic disease.	Sacituzumab govitecan is composed of an antibody targeted against Trop-2 coupled with a toxin known as SN-38 by an acid-labile hydrazone cleavable linker. Trop-2 is a transmembrane glycoprotein that is overexpressed in many solid tumors [94].
Loncastuximab Tesirine-Lpyl	Loncastuximab tesirine received accelerated approval in 2021 for patients with diffuse large B-cell lymphoma), after progressing on two or more lines of systemic therapy.	Loncastuximab tesirine is composed of an antibody against CD19, which is linked through a cleavable enzymatic type linker with SG3199, a cytotoxic alkylating agent. SG3199 is a synthetic pyrrolobenzodiazepine dimer that has a potent cytotoxic effect by promoting the formation of DNA interstrand cross-links and subsequently halting cell division [95,96].
Tisotumab Vedotin	In 2021, the FDA granted approval of tisotumab vedotin for adult patients with either recurrent or metastatic cervical cancer with disease progression, despite receiving chemotherapy.	This molecule is made up of a monoclonal antibody to target tissue factor (TF-011), a cleavable mc-VC-PABC linker and MMAE, the same cytotoxic agent used in brentuximab vedotin, polatuzumab vedotin, and enfortumab vedotin [97]

## Data Availability

No new data were created or analyzed in this study. Data sharing is not applicable to this article.

## Abbreviations

The following abbreviations are used in this manuscript:

IrAEs	Immune-related adverse events
ICIs	Immune cell inhibitors
FDA	Food and Drug Administration
EMA	European Medicines Agency
LC-MS	Liquid chromatography–mass spectrometry
LC-MS/MS	Liquid chromatography–tandem mass spectrometry
MRD	Measurable residual disease
LB	Liquid biopsy(biofluids)
MM	Multiple melanoma
MALDI-TOF MS	Matrix-assisted laser desorption ionization time-of-flight mass spectrometry
PTMs	Post-translational modifications
PD-1	Programmed death-1
PD-L1	Programmed death legend-1
TNBC	Triple-negative breast cancer
RFS	Recurrence-free survival
OS	Overall survival
IHC	Immunohistochemistry
GC	Gastric cancer
GBM	Glioblastoma Acute
AML	Acute myeloid leukemia
ADCs	Antibody–drug conjugates ADCs
ES-SCLC	Extensive-stage small-cell lung cancer
scFv	The single-chain variable fragment
NGS	Next-generation sequencing.
